# Association of p53 and mitochondrial gene with chemosensitization by metformin in ovarian cancer

**DOI:** 10.18632/oncotarget.22863

**Published:** 2017-12-02

**Authors:** De-Hua Wei, Yan Wang, Hui-Rong Shi

**Affiliations:** ^1^ Department of Gynecology, Puyang People's Hospital Affiliated to Xinxiang Medical University, Henan 457000, China; ^2^ Department of Gynecology, The First Affiliated Hospital of Zhengzhou University, Zhengzhou 450052, China

**Keywords:** ovarian cancer, metformin, p53, D-loop region, chemosensitivity

## Abstract

**Objective:**

This study aims to investigate the association of p53 and D-loop gene with drug resistance and sensitization induced by metformin in ovarian cancer.

**Results:**

Metformin suppresses cells in a time-dependent manner, but the inhibition does not change with the dose of metformin. This suggests that within a certain range of concentration in the body, metformin has a constant inhibitory effect on cells; and the long-term use of metformin yields a better effect.

**Conclusions:**

Metformin enhances the sensitivity of drug-resistant ovarian cancer cells to chemotherapy.

**Methods:**

The third passage cells of 17 cancerous ovarian tissues, which were successfully passaged five times, were used as the study objects; and the SKOV3 cell line was used as the positive control. After three adaptations, cells were cultured for 72 hours in an orthogonal experiment using drugs that were used for adaptation. Then, the inhibitory rate on cells in the experimental group was observed by CCK8 assay, in order to study the sensitization effect of metformin in different chemotherapies of ovarian cancer.

## INTRODUCTION

Ovarian cancer is one of the female genital tumors that has recently been found to have a high prevalence rate, which has no early symptoms and lacks an early diagnosis approach [1, 2]. Therefore, patients with this disease are often found to be at the middle and late stages at the first diagnosis [3]. Furthermore, the onset age of this disease has become younger [4]. Many experimental studies have revealed that metformin can inhibit the growth of tumor cell lines [3, 5–8]. Some studies have confirmed that [9–12] metformin could increase chemosensitivity, especially in combination with paclitaxel. Furthermore, it could significantly improve tumor cycle arrest, and delay tumor cell growth. In the process of apoptosis, mitochondria are a regulation object of p53 and an important participant in the energy metabolism in cells. The D-loop region is the region in the mitochondria where mutation most likely occurs. However, there are few studies on the relationship between gene mutations in these two and the sensitization of ovarian cancer. This study was designed to investigate the association of gene polymorphisms in the p53 gene and mitochondrial genome D-loop region with metformin-induced sensitization and prognosis of ovarian cancer.

## RESULTS

### Adaptation for the drug resistance and drug sensitivity test of ovarian cancer cells

The cell inhibition rate was used to evaluate the inhibitory effect of drugs on the tumor and the effect of adaptation for drug resistance.

In the detection of the effect of the combined use of drugs, the results were calculated using the following procedures: the average detection value of CCK8 for the blank controls was subtracted from the average detection value of the CCK8 of the A–D lines of each column. Then, the results were divided by the average detection value of CCK8 for the blank controls. The final results were considered as the cell inhibitory rate under drug action for a short time.

In the detection of the effect of artificial adaptation for resistance, these results were calculated using the following procedures: the average detection value of CCK8 for blank controls was subtracted from the average detection value of CCK8 for the E-H lines of the H1 column. Then, the results were divided by the average detection value of CCK8 for blank controls.

### Inhibition of metformin on ovarian cancer cells and non-cancer ovarian cells

The inhibition rate in this experiment was termed as follows: the average detection value of CCK8 for the 1–4 or 5–8 columns of each line was subtracted from the average detection value of CCK8 for blank controls. Then, these results were divided by the average detection value of CCK8 for blank controls. In all 17 ovarian cancer cell lines and non-cancer ovarian cells, an inhibition rate of 2.72–3.56% within 72 hours after treatment and an inhibition rate of 3.24–6.83% within 120 hours after treatment were observed. Metformin inhibited cells in a time-dependent manner, but the inhibition did not change with the dose of metformin. This suggests that metformin has a constant inhibitory effect on cells within a certain range of concentration in the body.

## DISCUSSION

The initial exploration on the association between metformin and cancer is a epidemiological study that revealed higher incidence of cancer in diabetes mellitus patients than other populations, hence it was speculated that use of metformin in diabetes patients increased the risk of developing tumors. Subsequently, in order to verify the hypothesis, some scholars investigated the difference in cancer incidence and mortality among diabetics taking metformin and other antidiabetic drugs, and Evans proposed in 2005 that metformin not only does not increase cancer risk, but reduces it. In addition, the risk of cancer in metformin users was inversely proportional to the duration of metformin medication and the dose of metformin. Libby came up with a similar conclusion in 2009. Although these are only based on the hypothesis that the two are associated, which is based on retrospective studies, it still excites tumor researchers. Subsequently, many independent institutions and teams studied this and got similar conclusions, which promoted the study on the application of metformin in cancer therapy. In the present study, the concentration of the drug was particularly adjusted to the clinical concentration. The anti-tumor effect of metformin was confirmed by *in vitro* cytology test. In a study conducted by Hadad S *et al.*, before surgery 47 diagnosed patients with breast cancer were randomly assigned to different groups and received oral administration of metformin for a period of time, results revealed that metformin effectively reduced the Ki67 positive cell rate in patients with breast lesions, suggesting that metformin can prolong survival in patients with breast cancer. Bonanni B *et al.* added detection of insulin levels in a similar study, results revealed that metformin lowered insulin levels, and patients with low insulin levels had better outcomes than those with high insulin levels. For experimental objects, we used the cancerous tissues of 30 patients with ovarian cancer as the research objects. In our study it was found that some cells were not sensitive to metformin, and in some cells the effect of sensitization was remarkable, when metformin was used combined with chemotherapeutic drugs, it could allow low concentrations of chemotherapy drugs to function as high concentrations of chemotherapeutic drugs, which is of important clinical significance.

At present, it is clinically often recommended to use the combined therapy of low dose AUC and paclitaxel to reduce blood toxicity [13]. For patients with middle or advanced ovarian cancer, who have long been treated with chemotherapy, at least 80% of these patients would develop secondary drug resistance at different levels; and the 5-year survival rate would be hovering at 20–30%. At present, the theoretical system of present studies cannot completely explain the phenomena of the incidence, development and drug resistance of ovarian cancer [14]. Therefore, there is an urgent need to study the mechanism of the occurrence, development and drug resistance of ovarian cancer, and develop different treatment strategies directed at different drug resistance, in order to improve the survival rate and quality of life of patients with ovarian cancer. Most studies have revealed that metformin could reduce the risk of malignancy by 23%, and the effect was significantly related to the dose of metformin [15]. Specific for its effect on ovarian cancer, a study revealed that [16] the administration of metformin could significantly improve the survival time of ovarian cancer patients. In 2012, *Cell* [17] and *Nature* [18] published some studies, which revealed that metformin could reduce the growth, proliferation, apoptosis and invasion of tumors by altering the cell cycle *via* the AMPK pathway. Furthermore, Rattan *et al.* [19] revealed in a study on ovarian cancer that metformin could suppress the growth of ovarian cancer cells. In order to clinically use metformin to prevent or treat ovarian cancer, the dose of metformin must be determined through experiments. In the present study, primary culture and the artificial induction of the resistance of ovarian tissue were adopted. The results of the experiments on 17 cells revealed that differences in the chemotherapy sensitivity and sensitization effect of metformin on different cells were statistically significant.

## CONCLUSIONS

Metformin has a chemotherapeutic effect on drug-resistant ovarian cancer cells. The sensitization effect of metformin in 30 ovarian cancer patients was investigated, it is confirmed that metformin has obvious sensitization effect in most ovarian cancer cells, in addition, we screened the potential indicators for forecasting of metformin sensitivity using p53 and mitochondrial D-loop region gene polymorphism. Exon4-72G + D-loop309T in cells can be used as a potential detection indicator for drug resistance, and Exon6-198G + Exon8-67A + D-loop309T + D-loop446A can be used as potential indicators for metformin sensitivity.

## MATERIALS AND METHODS

### Experimental materials

#### Clinical specimens

The peripheral blood and tissues used in this study were sterilely collected before surgery or during the first ovarian cancer cytoreductive surgery from 30 patients who were treated at the First Affiliated Hospital of Zhengzhou University (FAH-ZU) and Puyang People’s Hospital from October 2013 to January 2014.

In addition, non-cancerous ovarian tissues and peripheral blood specimens were collected from 15 patients who underwent ovariectomy at FAH-ZU and Puyang People’s Hospital from August 2013 to September 2014, and were used as negative controls. The tissue specimens underwent primary cell culture once obtained.

### Experimental methods

#### Acquisition of ovarian tissue cells from non-cancer patients

#### Sampling

Sixty patients who were admitted at FAH-ZU and Puyang People’s Hospital, and resided in Henan province for more than 20 years, were selected as the study subjects.

#### Culture

(1) Cell separation was performed by centrifugation.

(2) A three times volume of 0.3% collagen enzyme was added to the sedimented cell mass, and reacted in a water bath at 37°C for 15–20 minutes. Then, cells were re-suspended with RPMI 1640 medium containing 10% fetal bovine serum preheated in a water bath at 37°C.

(4) Living cells were counted and cultured for one hour, taken out, and centrifuged.

(5) The sediments were added with 0.3% collagen enzyme solution and M199 culture medium (SLM) containing 5% fetal bovine serum and 1% OEpiCGS (cat7352) for culture.

(6) Every two days, half of the volume of the M199 (SLM) medium containing 5% fetal bovine serum and 1% OEpiCGS was replaced until cells covered 70% of the bottom area of the culture flask.

#### Acquisition of ovarian cancer tissue cells

#### Acquisition

Patients who underwent initial ovarian cancer cytoreductive surgery in FAH-ZU and Puyang People’s Hospital from October 2013 to January 2014, and resided in Henan province for more than 20 years, were selected as the study subjects. DNA was extracted from sterilely obtained cancerous ovarian tissues using a paraffin section gene extraction kit.

#### Cultivation

(1) The ovarian cancer tissue was cut into small granules, flushed into a centrifuge tube with sterile phosphate buffered saline (PBS), centrifuged at 2000 rpm and 4°C for five minutes. The supernatant was discarded.

(2) The remaining solution was digested with collagenase solution, filtered, added with 1 volume of 0.25% trypsin solution, digested in a water bath at 37°C for 10 minutes, and filtered with sterilized glass wool.

Cells were re-suspended with a small amount of RPMI 1640 medium containing 20% fetal bovine serum, and were counted according to the method described in section 1.2.1.2.

(4) After the counting of living cells, the solution was diluted with 1640 medium containing 10% fetal bovine serum to a concentration of 5 × 10^6^/ml, inoculated in a 25-cm^2^ cell culture bottle, and placed in a cell incubator with 5% CO_2_ at 37°C for culture.

(5) After one hour of culture, the medium was taken out, poured into a new centrifuge tube for centrifugation, and added with medium for culture.

(6) At 48 hours of culture, the medium was discarded. Then, the cell surface was washed with sterile PBS, and the supernatant was discarded. This washing was conducted three times.

### Resistance adaptation and drug sensitivity test of ovarian cancer cells

#### Preparation of the work solutions of metformin, paclitaxel and carboplatin

The effects of these three alone and their combined use on ovarian cells were observed by simulating normal blood medication levels of the body *in vitro*. The doses of metformin, carboplatin and paclitaxel were designed as three levels of free, low concentration, and high concentration, respectively. According to the principle of orthogonal experiments, the volumes of the drug liquid were assigned based on three factors and their three levels, as shows in Table 1.

**Table 1:** Experimental grouping L9 (3^3^ )

Factor Group	Metformin (A)	Carboplatin (B)	Paclitaxel (C)
H1	Level 1 (0)	Level 1 (0)	Level 1 (0)
H2	Level 1 (0)	Level 2 (10 μg/mL)	Level 2 (0.6 μg/mL)
H3	Level 1 (0)	Level 3 (2 5 μg/mL)	Level 3 (1.2 μg/mL)
H4	Level 2 (0.5 μg/mL)	Level 1 (0)	Level 2 (0.6 μg/mL)
H5	Level 2 (0.5 μg/mL)	Level 2 (10 μg/mL)	Level 3 (1.2 μg/mL)
H6	Level 2 (0.5 μg/mL)	Level 3 (25 μg/mL)	Level 1 (0)
H7	Level 3 (2.5 μg/mL)	Level 1 (0)	Level 3 (1.2 μg/mL)
H8	Level 3 (2.5 μg/mL)	Level 2 (10 μg/mL)	Level 1 (0)
H9	Level 3 (2.5 μg/mL)	Level 3 (25 μg/mL)	Level 2 (0.6 μg/mL)

The three mother liquors were mixed with M199 (SLM) medium containing 5% fetal bovine serum and 1% OEpiCGS, in order to prepare into the mixed liquid of nine concentrations required by the orthogonal experiment. These were labeled as H1-9 for future use, according to the order of the orthogonal table. In addition, the metformin was prepared into 2.5 μg/ml and 0.5 μg/ml work solutions, noted as T1 and T2, respectively. The mother liquor of carboplatin was added with the M199 (SLM) medium containing 5% fetal bovine serum and 1% OEpiCGS until a volume of 25 μg/ml (P1) was reached, and the mother liquor of paclitaxel was added with M199 (SLM) medium containing 5% fetal bovine serum and 1% OEpiCGS until a volume of 1.2 μg/ml (P2) was reached.

Then, 400 μL of the above solutions were respectively coated on aseptic agar plates and cultured at 37°C for five days for the sterility test.

#### Preparation of experimental cells

(1) Positive control: The SKOV3 cell line was used as the positive control. A frozen storage tube containing SKOV3 tumor cells was taken out from the liquid nitrogen tank, rapidly placed in a water bath with a constant temperature of 37°C, and shook frequently to allow the cryopreserved cells to thaw within one minute. Then, the cell suspension in the frozen storage tube was sucked out with a pipette on a clean bench, and injected into a centrifuge tube previously filled with M199 (SLM) medium containing 5% fetal bovine serum. The cover of the centrifuge tube was closed, and the suspension was centrifuged at 2,000 rpm for five minutes. Next, the supernatant was discarded, 5 ml of M199 (SLM) medium containing 5% fetal bovine serum was added, and centrifuged again. Cells were re-suspended with the appropriate amount of M199 (SLM) medium containing 5% fetal bovine serum and 1% OEpiCGS, the cell suspension was placed in the culture bottle, and cells were incubated in an incubator at 37°C with 5% CO_2_. The medium was replaced on the next day to remove the floating dead cells. When tumor cells grew to the logarithmic growth phase (that is, the cells covered approximately 80% of the bottom area of the bottle), cells were passaged. In the third generation, cells were digested with 0.3% of collagenase and added with M199 (SLM) medium containing 5% fetal bovine serum and 1% OEpiCGS to dilute to 5 × 10^5^/ml.

(2) Experimental group: Ovarian cancer cells in the third generation derived from the 17 primary cells were used as the experimental subjects. Cells were digested with 0.3% collagenase and added with M199 (SLM) medium containing 5% fetal bovine serum and 1% OEpiCGS to dilute to 5 × 10^5^/ml.

#### Artificial adaptation of ovarian cancer cells

(1) Cells of the third generation were digested with 0.3% collagenase, and were added with M199 (SLM) medium containing 5% fetal bovine serum and 1% OEpiCGS, in order to dilute into the two concentrations of 5 × 10^5^/ml and 1 × 10^5^/ml. Cell suspension at a concentration of 5 × 10^5^/ml was added to 48 wells in the A–D lines of the 96-well cell culture plate (Table 1.4), and the cell suspension at a concentration of 1 × 10^5^/ml was added to 48 wells in the E-H lines. The addition amount was 100 μL for each well. After sample loading, cells were placed in a cell incubator and cultured with 5% CO_2_ at 37°C for 24 hours.

(2) For each well, 100 μL of prepared drug liquid was added according to the orders and positions shown in Table 2. After the drug liquid was added, cells were placed in a cell incubator to culture with 5% CO_2_ at 37°C for one hour. Then, the drugs were sucked out, added with M199 (SLM) medium containing 5% fetal bovine serum and 1% OEpiCGS, and cultured until 70% of the bottom area was covered by cells at a cell density of 5 × 10^5^/mL in more than 50% of the wells.

**Table 2:** The table of addition of sample in 96 cell culture plate

	Column number											
Line number	1	2	3	4	5	6	7	8	9	10	11	12
A	H1	H2	H3	H4	H5	H6	H7	H8	H9	T1	P1	P2
B	H1	H2	H3	H4	H5	H6	H7	H8	H9	T1	P1	P2
C	H1	H2	H3	H4	H5	H6	H7	H8	H9	T1	P1	P2
D	H1	H2	H3	H4	H5	H6	H7	H8	H9	T1	P1	P2
E	H1	H2	H3	H4	H5	H6	H7	H8	H9	T1	P1	P2
F	H1	H2	H3	H4	H5	H6	H7	H8	H9	T1	P1	P2
G	H1	H2	H3	H4	H5	H6	H7	H8	H9	T1	P1	P2
H	H1	H2	H3	H4	H5	H6	H7	H8	H9	T1	P1	P2

(3) The culture medium in the plate was sucked out, and 120 μL of the prepared drug liquid was added into each well according to the orders and positions shown in Table 2. After the drug liquid was added, cells were cultured in an incubator with 5% CO_2_ at 37°C for one hour. Then, the drug was sucked out, and M199 (SLM) medium containing 5% fetal bovine serum and 1% OEpiCGS was added to culture, until 70% of the bottom area was covered with cells in more than 30% of the wells in the A-D lines of each plate.

(4) The procedure in step 3 was repeated once.

#### Sensitization of metformin on the chemotherapy-resistant ovarian cells

(1) Cells that were adapted for drug resistance three times (as described in section 1.2.4.3) were observed daily under a microscope. When approximately 70% of the bottom area was covered with cells in more than three wells in each 96-well plate, the culture medium in the whole plate was sucked out.

(2) For each well, 120 μL of the prepared drug liquid was added to the wells in the A–D lines of the 96-well plate according to the orders and positions shown in Table 1, and cells were cultured for 72 hours.

(3) For each well, 120 μL of M199 (SLM) medium containing 5% fetal bovine serum and 1% OEpiCGS was added to wells in the E–H lines of the 96-well plate, and cells were cultured for 72 hours.

(4) For detection, 12 μL of CCK8 was added to each well. Then, cells were placed in a cell incubator to culture with 5% CO_2_ at 37°C for two hours, and the optical density (OD) value at 450 nm was detected using an enzyme micro-plate reader.

#### The inhibitory effect of metformin on the growth of ovarian cells

Ovarian cells from non-cancer patients were passaged until the third generation, and these cells were digested by the method described in Section 1.2.4.3. On the 96-well plate, one type of cell was added to one line, each plate could be added with eight types of cells, and cells were added to another four 96-well plate according to the same position and order. At one day of culture, the culture medium in the plates was sucked out, 120 μL of T1, T2 and blank control (M199 medium containing 15% fetal bovine serum and 1% OEpiCGS) were added to each 96-well plate, and cells were cultured at 37°C. Among these, T1 was added to the first four columns of each plate, T2 was added to the middle four columns of each plate, and the medium was added to the last four columns of each plate to serve as the blank control. During the culture, a plate was taken out for CCK8 detection every 24 hours.

## Abbreviations

FAH-ZU: First Affiliated Hospital of Zhengzhou University
PBS: phosphate buffered saline
